# METTL3 promotes osteogenic differentiation of human umbilical cord mesenchymal stem cells by up-regulating m6A modification of circCTTN

**DOI:** 10.1042/BSR20231186

**Published:** 2024-03-12

**Authors:** Shujiang Chen, Xiaoqiong Duan, Yanjin He, Wenchuan Chen

**Affiliations:** 1 State Key Laboratory of Oral Diseases and National Clinical Research Center for Oral Diseases, West China school of Stomatology, Sichuan University, Chengdu, Sichuan, China; 2 Department of Prosthodontics, West China Hospital of Stomatology, Sichuan University, Chengdu, Sichuan, China; 3 Institute of Blood Transfusion, Chinese Academy of Medical Sciences and Peking Union Medical College, Chengdu, Sichuan, China

**Keywords:** circCTTN, human umbilical cord mesenchymal stromal cells, m6A, METTL3, osteogenic differentiation

## Abstract

Background: Human umbilical cord mesenchymal stem cells (hUCMSCs) are promising seed cells in bone tissue engineering. circRNA and N6-methyladenosine (m6A) RNA methylation play important roles in osteogenic differentiation. Here, we investigated the potential relevance of a critical circRNA, hsa_circ_0003376 (circCTTN), and methyltransferase-like 3 (METTL3) in osteogenic differentiation of hUCMSCs.

Methods: Expression of circCTTN after hUCMSC osteogenic induction was detected by qRT-PCR. Three databases (RMBase v2.0, BERMP, and SRAMP) were used to predict m6A sites of circCTTN. RNA was enriched by methylated RNA immunoprecipitation (MeRIP), followed by quantitative real-time polymerase chain reaction to detect m6A level of circCTTN after METTL3 overexpression and osteogenic induction. RNA pull-down, Western blotting, and protein mass spectrometry were performed to investigate the potential mechanisms by which METTL3 promoted m6A modification of circCTTN. Bioinformatic analyses based on database (STRING) search and co-immunoprecipitation were used to analyze the proteins that interacted with METTL3.

Results: Overexpression of METTL3 promoted osteogenic differentiation of hUCMSCs and increased m6A level of circCTTN. Two potential m6A modification sites of circCTTN were predicted. No direct interaction between METTL3 and circCTTN was observed. Thirty-one proteins were pulled down by probes specific for circCTTN, including NOP2, and two m6A reading proteins, EIF3A and SND1. Bioinformatics analysis and co-immunoprecipitation showed that METTL3 interacted with EIF3A indirectly through NOP2.

Conclusions: METTL3 promotes the osteogenic differentiation of hUCMSCs by increasing the m6A level of circCTTN. However, METTL3 does not bind directly to circCTTN. METTL3 interacts with circCTTN indirectly through NOP2 and EIF3A.

## Introduction

Although bone marrow mesenchymal stem cells (BMSCs) have long been regarded as the gold standard seed cells in bone tissue engineering [1], they have some limitations, such as invasive procedures during acquisition, limited number of cells collected [2], dependence on the donor’s age [3,4], and physiological conditions [5,6]. Instead, human umbilical cord mesenchymal stem cells (hUCMSCs), isolated from Wharton’s jelly [7], exhibit strong proliferation and differentiation [7–9]. Previous studies have demonstrated that hUCMSCs have similar osteogenic ability to BMSCs [10–13]. Therefore, hUCMSCs have the potential to replace hBMSCs as seed cells for bone tissue engineering. However, the detailed mechanisms of osteogenic directional differentiation of hUCMSCs have not been well studied.

Circular RNA (circRNA) is noncoding RNA that participates in various pathophysiological processes [14–18]. Many studies have found that a variety of circRNAs have N6-methyladenosine (m6A) modification sites [19]. m6A modification of specific circRNAs affects many pathophysiological processes, such as cell differentiation, immune defense, and development of tumors and other diseases [20,21]. Nice example comes from METTL3 (methyltransferase-like 3), which facilitates m6A methylation of circ_0008542, to initiate osteoclast bone resorption [22].

m6A is the most prevalent internal modification in mammalian RNA, and the process is precisely regulated by m6A writers, erasers, and readers [23–25]. m6A writer is a large methyltransferase complex consisting of METTL3, METTL14, VIRMA (Vir-like m6A methyltransferase associated), and Wilms’ tumor 1-associated protein (WTAP), which mediates m6A methylation on adenosine of target RNAs [26–28]. Numerous studies have shown that m6A modification plays an important role in cell differentiation. Bertero et al. reported that m6A modification affected cell pluripotency and differentiation [29]. As one of the m6A writers, METTL3 was positively correlated with osteogenic differentiation and negatively correlated with adipogenic differentiation of BMSCs. Osteogenic differentiation of mouse BMSCs was significantly inhibited following selective knockout of METTL3, indicating a close link between m6A modification and METTL3 expression [30]. The deletion of METTL3 in porcine BMSCs could promote adipogenesis [31].

Our previous transcriptomic profiling study of hUCMSCs during osteogenic differentiation demonstrated that circ_0003376 (circCTTN) was significantly increased [32]. To understand the biological function of circCTTN in osteogenic differentiation, we detected the m6A levels of circCTTN in hUCMSCs following osteogenic induction using RNA immunoprecipitation assay (MeRIP-qPCR). We identified that m6A levels of circCTTN at two predicted modification sites were both significantly increased in hUCMSCs after osteogenic induction, and one specific methyltransferase, METTL3, contributed to this increased methylation. We also performed some underlying mechanistic studies and demonstrated that circCTTN did not interact with METTL3 directly. Instead, METTL3 might have interacted with a m6A reader proteins eukaryotic translation initiation factor 3 subunit A (EIF3A) through nucleolar protein 2 (NOP2) protein, which were both pulled down by probes specific for circCTTN.

Our present study revealed a novel regulatory mechanism of m6A modification of one specific circRNA to promote osteogenic differentiation of hUCMSCs.

## Materials and methods

### Cell culture and osteogenic differentiation of hUCMSCs

The hUCMSCs (Jennio Biological Technology, Guangzhou, China) were cultured in complete medium, containing Dulbecco’s minimum essential medium (Gibco, U.S.A.), 10% fetal bovine serum (Gibco), and 1% penicillin/streptomycin. The cells were incubated at 37°C with 5% CO_2_ in a humidified incubator and the medium was replaced every other day. Fourth-passage hUCMSCs were used for osteogenic induction. The osteogenic induction medium consisted of complete Dulbecco’s minimal essential medium, dexamethasone (0.1 mM), ascorbic acid (50 μg/ml), and β-glycerolphosphate (10 mM) (all from Sigma-Aldrich) [33]. hUCMSCs with 70–80% confluence were cultured in the osteogenic induction medium, which was replaced every other day.

### Prediction of potential m6A modification sites of circCTTN

RMBase v2.0 (http://rna.sysu.edu.cn/rmbase/) [34], BERMP (http://www.bioinfogo.org/bermp) [35], and SRAMP (http://www.cuilab.cn/sramp/) [36] were used to predict the potential m6A modification sites of circCTTN.

### Overexpression of METTL3

The expression vector carrying METTL3 gene was constructed using recombinant adenovirus vector (Hanbio, China) and transfected into hUCMSCs as the experimental group (OE-METTL3 group, *n*=3). Cells cultured in complete medium without transfection served as the blank control group (*n*=3). Inverted fluorescence microscopy was used to observe the transfection efficiency. After transfection, the cells in two groups were incubated in the osteogenic induction medium for 7 days for quantitative real-time polymerase chain reaction (qRT-PCR), Western blotting, alkaline phosphatase (ALP) staining, MeRIP-qPCR, RNA pulldown, and co-immunoprecipitation (Co-IP).

### RNA extraction and qRT-PCR

TRIzol (Invitrogen, U.S.A.) was used to extract total RNA from hUCMSCs at 7 days following osteogenic induction. The quantity and quality of total RNAs were assessed by NanoDrop ND-1000 (NanoDrop, U.S.A.). Only RNA samples with absorbance 260/280 ratios between 1.8 and 2.0 were used. After mixing RNA samples (8 µl) with Mix (4 µl, ReverTre Ace qPCR RT Master Mix; Toyobo) and diethyl pyrocarbonate (DEPC) water, the mixture (20 µl) was incubated at 37°C for 15 min, 50°C for 5 min and 98°C for 5 min to perform reverse transcription. The resultant cDNA (5 µl) samples were mixed with 2×Novosta SYBR qPCR SuperMix (Novoprotein) (10 µl), forward (F) (1 µl)/reverse (R) (1 µl) primers (Table 1) and RNase-free distilled water (3 µl) to perform qPCR. ACTB and GAPDH were used as the internal control genes, and the PCR procedure is shown in Table 2. Melting curve analysis was performed to confirm nonspecific amplification. The relative expression levels of circCTTN were calculated using the ΔΔ*C*t method after being normalized to ACTB, and the relative expression levels of osteogenesis-related genes, ALP, COL1, RUNX2, and METTL3 were calculated using the ΔΔ*C*t method after being normalized to GAPDH.

**Table 1:** Forward (F)/Reverse (R) primer sequences for qRT-PCR

Gene	Primer sequence
COL1	F: 5′-CTGGTCATCCTGGGAAAGAA-3′
	R: 5′-TTGAATCCTGGAAAGCCATC-3′
ALP	F: 5′-AGCACTCCCACTTCATCTGGAA-3′
	R: 5′-GAGACCCAATAGGTAGTCCACATTG-3′
RUNX2	F: 5′-TCCACACCATTAGGGACCATC-3′
	R: 5′-TGCTAATGCTTCGTGTTTCCA-3′
METTL3	F: 5′-TTGTCTCCAACCTTCCGTAGT-3′
	R: 5′-CCAGATCAGAGAGGTGGTGTAG-3′
GAPDH	F: 5′-GGCCTCCAAGGAGTAAGACC-3′
	R: 5′-AGGGGAGATTCAGTGTGGTG-3′
circ_0003376	F: 5′-GGAGACCGACCCTGATTTTG-3′
	R: 5′-CGCTTGGAGATAAAAGGCTGT-3′
ACTB	F: 5′-GTGGATCAGCAAGCAGGAGT-3′
	R: 5′-AAAGCCATGCCAATCTCATC-3′

**Table 2:** qRT-PCR procedure

Program	Time	Temperature (°C)	Cycle number
Pre-denaturation	10 min	95	1
Denaturation	10 s	95	
Annealing and extension	60 s	60	40
Dissolution curve	10 s	95	1
	60 s	60	1
	15 s	95	1
	15 s	60	1

### Western blotting

Western blotting was used to confirm expression of osteogenesis-related genes (ALP, COL1, and RUNX2) and overexpression of METTL3 protein. Total proteins were extracted by Radio Immunoprecipitation Assay Lysis Buffer (Beyotime, China) and quantified by an enhanced BCA Protein Assay Kit (Beyotime). Equal amounts of protein (30 µg) were resolved through 10% SDS-PAGE (Epizyme Biotech, China) before they were transferred to polyvinylidene difluoride membranes (Millipore, U.S.A.). Bovine serum albumin (5%; BIOFROXX, German) was used to block membranes. The protein bands were immunoblotted with specific primary antibodies: rabbit anti-ALP (1:1000 dilution, Huabio, China), rabbit anti-COL1 (1:1000 dilution, Huabio, China), rabbit anti-RUNX2 (1:1000 dilution, Huabio), rabbit anti-METTL3 (1:1000 dilution, Huabio), and rabbit anti- GAPDH (1:1000 dilution, Huabio). After incubation with goat anti-rabbit secondary antibody (1:1000 dilution, SAB), the membranes were scanned using Bio-Rad ChemiDoc MP.

### ALP staining

The cells of three groups after differentiation for 7 days were stained by an ALP staining kit (Beyotime) with nitro-blue tetrazolium chloride and 5-bromo-4-chloro-3′-indolyphosphate p-toluidine salt (NBT/BCIP) solution at room temperature for 30 min and washed by double-distilled water. The results for the three groups were observed under a microscope according to the depth of staining.

### MeRIP-qPCR

MeRIP-qPCR was performed by Cloudseq Biotech Inc. (Shanghai, China) with GenSeq m6A MeRIP Kit. RNA was isolated and fragmented into lengths of ≤200 nucleotides. Fragmented RNAs (3μg) were saved as input control for MeRIP-qPCR. The RNA samples were immunoprecipitated with magnetic beads precoated with anti-m6A antibody (Abcam, U.S.A.). The m6A-modified RNA fragments were eluted and purified for qRT-PCR analysis. The m6A levels of predicted circCTTN m6A modification sites were measured by qRT-PCR with sequence-specific primers (Life Technologies, Shanghai, China) designed for the predicted sites (Table 3).

**Table 3:** Primer sequences of predicted sites for qRT-PCR

Site	Primer sequence
circCTTN-54	F: 5′-AATCAGTCCCCAATGCCTGG-3′
	R: 5′-GTCATCCTGGGCGATGGAC-3′
circCTTN-159	F: 5′-AGCGGAAAGAAAGATGTGGA-3′
	R: 5′-CAAAATCAGGGTCGGTCTCC-3′

### RNA pulldown

RNA pulldown was performed by Cloudseq Biotech (Shanghai, China) with GenSeq RNA Pull Down. A positive circCTTN probe (5′-biotin-CCAGGCATTGGGGACTGATTCCGTTTGATATGCTCCTGGTGCCCGGAG-3′) and negative probe (5′-biotin -CTCCGGGCACCAGGAGCATATCAAACGGAATCAGTCCCCAATGCCTGG- 3′) were synthesized by Life Technologies. After biotinylation, the probes were incubated with the beads at room temperature for 1 h for immobilization. The biotinylated beads were incubated with osteogenesis-induced hUCMSCs cell lysate at room temperature for 2 h. The biotinylated beads were magnetically separated and washed five times. METTL3 was detected by Western blotting and the unknown proteins were identified by Easy nLC 1000 system (ThermoFisher Scientific, U.S.A.) and Q-Exactive mass spectrometer (ThermoFisher Scientific). STRING database (https://cn.string-db.org/) was used to identify potential protein interactions [37].

### Co-IP

Co-IP was performed by Cloudseq Biotech. The cells were lysed to extract protein by Radio Immunoprecipitation Assay Lysis Buffer (Beyotime) and quantified by an enhanced BCA Protein Assay Kit (Beyotime). Supernatant (20 μl) from the cell lysis was boiled with 5× loading buffer (20 μl) was used as input control for Co-IP. The protein samples were immunoprecipitated with magnetic beads precoated with anti-EIF3A (Huabio) and anti-METTL3 (Proteintech, China) antibody. After that, the proteins binding with beads were eluted and used as the IP group for Western blotting. The protein bands were immunoblotted with specific primary antibodies: rabbit anti-NOP2 (1:1000 dilution, Huabio) and rabbit anti-GAPDH (1:1000 dilution, Huabio). After incubation with goat anti-rabbit secondary antibody (1:1000 dilution, SAB), the membranes were scanned using Bio-rad ChemiDoc MP.

### Statistical analysis and graphics

Experiments were repeated three times independently. Statistical analysis was performed using GraphPad Prism 8. All group data were presented as means ± standard deviation. Student's *t* test was used for statistical analysis, and *P*<0.05 was considered statistically significant (*P<0.05, **P<0.01, ***P<0.001, and ****P<0.0001. NS; not statistically significant).

## Results

### METTL3 overexpression promotes osteogenic differentiation of hUCMSCs

**Figure 1:**
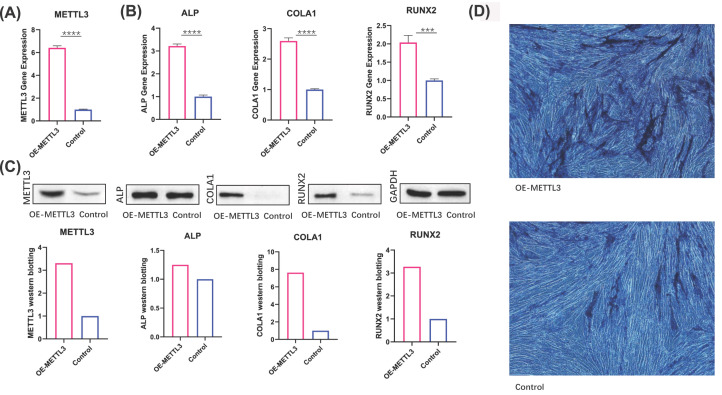
METTL3 promotes osteogenic differentiation of hUCMSCs hUCMSCs were transfected by recombinant adenovirus carrying METTL3 and osteogenesis was induced. (**A**) levels of METTL3 was analyzed by qRT-PCR at 24 h (OE-METTL3 vs. Control: P<0.0001). (**B**) Expression of three osteogenesis-related genes was analyzed by qRT-PCR at 7 days (ALP: OE-METTL3 vs. Control: *P*<0.0001; COL1: OE-METTL3 vs. Control: *P*<0.0001; RUNX2: OE-METTL3 vs. Control: *P*<0.001). (**C**) Expression of METTL3 and three osteogenesis-related genes was analyzed by Western blotting at 7 days. (**D**) ALP staining under microscopy (OE-METTL3: METTL3-overexpressed hUCMSCs; Control: cultured hUCMSCs only. *P<0.05, **P<0.01, ***P<0.001, and ****P<0.0001. NS; not statistically significant).

We overexpressed METTL3 to identify its impact on cell osteogenic differentiation and the circCTTN m6A level (Figure 1). METTL3 was overexpressed by recombinant adenovirus and its effect on osteogenic differentiation of hUCMSCs was evaluated by qRT-PCR and Western blotting. Expression of METTL3 was up-regulated in hUCMSCs following recombinant adenovirus transfection at both mRNA (Figure 1A) and protein (Figure 1C) levels. To identify the effect of METTL3 overexpression on osteogenic differentiation of hUCMSCs, we analyzed expression of three osteogenesis-related genes by qRT-PCR and Western blotting. Expression of ALP, COL1, and RUNX2 was significantly increased in METTL3-overexpressed hUCMSCs compared with the control group (Figure 1B,C). ALP staining of hUCMSCs after transfection and osteogenic induction for 7 days are shown in Figure 1D (Supplementary Material S1). These results indicated that METTL3 overexpression stimulated osteogenic differentiation of hUCMSCs.

### METTL3 overexpression increased expression and m6A levels of circCTTN

**Figure 2:**
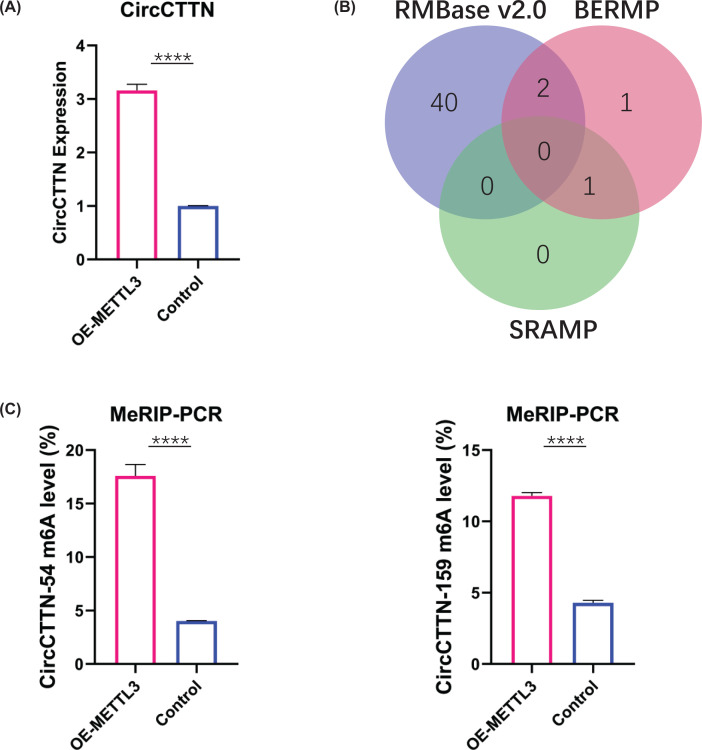
METTL3 overexpression increased expression and m6A levels of circCTTN in osteogenic differentiation of hUCMSCs (**A**) Expression of circCTTN was analyzed by qRT-PCR at 7 days in transfected and osteogenesis-induced hUCMSCs (OE-METTL3 vs. Control: *P*<0.0001). (**B**) Potential m6A modification sites of circCTTN were predicted from RMBase v2.0, BERMP and SRAMP. (**C**) m6A levels of circCTTN and potential m6A modification sites were analyzed by MeRIP-PCR at 7 days in transfected and osteogenesis-induced hUCMSCs (circCTTN-54: OE-METTL3 vs. Control: *P*<0.0001; circCTTN-159: OE-METTL3 vs. Control: *P*<0.0001). ( *P<0.05, **P<0.01, ***P<0.001, and ****P<0.0001. NS; not statistically significant).

In our previous study, circCTTN was significantly increased in hUCMSCs after osteogenic differentiation [32]. Here, we investigated whether up-regulation of circCTTN was relevant to METTL3. We overexpressed METTL3 in osteogenic-induced hUCMSCs and analyzed expression of circCTTN. We found a significant increase in circCTTN expression in METTL3-overexpressing hUCMSCs (OE-METTL3) (Figure 2A) (Supplementary Material S2).

Considering that METTL3 is a predominant catalytic enzyme in the m6A methyltransferase system, we investigated whether METTL3 affected m6A levels of circCTTN. We used RMBase v2.0, BERMP and SRAMP to predict the possible m6A sites of circCTTN, and 44 potential sites related to CTTN gene were obtained (Supplementary Material S3). Of these 44 sites, hsa_circ_0003376-54 (circCTTN-54) and hsa_circ_0003376-159 (circCTTN-159) were chosen for further study, as they overlapped in two databases (Figure 2B). MeRIP and qRT-PCR detected that m6A levels of these two m6A sites were significantly up-regulated in the METTL3-overexpressing group (Figure 2C) (Supplementary Material S4). These results indicate that METTL3 stimulates expression of circCTTN and up-regulates m6A levels of circCTTN.

### METTL3 up-regulates circCTTN m6A modification possibly through m6A readers, EIF3A, and RNA-methyltransferase family, NOP2

**Figure 3:**
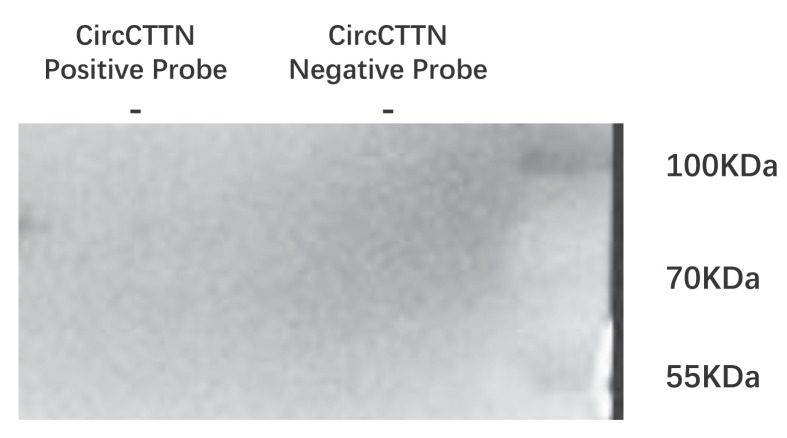
RNA pulldown and western blotting analysis of the interaction between circCTTN and METTL3 at 7 days after osteogenic induction in hUCMSCs

We investigated how METTL3 regulated circCTTN m6A levels. After hUCMSC osteogenic induction, RNA pulldown was performed using a biotin-labeled probe targeting circCTTN, followed by Western blotting of METTL3. We did not observe a direct interaction between circCTTN and METTL3 (Figure 3). To explore the potential proteins that were involved in the modification of circCTTN, we used mass spectrometry to identify those proteins that were pulled down by circCTTN probe. Thirty-one proteins were pulled down by probes specific for circCTTN, and they were up-regulated significantly compared with the negative control probe group (*P*<0.05) (Table 4) (Supplementary Material S5). Twelve of the 31 proteins were up-regulated <5 times, three were up-regulated 5–10 times, and 16 were up-regulated 20–50 times. Two of the 31 proteins were m6A readers, namely EIF3A and SND1 (Staphylococcal nuclease domain-containing protein 1), and was RNA-methyltransferase family, NOP2. Expression of EIF3A and SND1 was up-regulated by 3.6 and 4.1 times in the positive probe group, respectively. NOP2 was up-regulated by 2.2 times.

**Table 4:** Mass spectrometry of potential proteins pulled down by probes specific for circCTTN, which were up-regulated significantly compared with the negative probe group (*P*<0.05)

Protein names	Fold change	Protein names	Fold change
NOP2	2.180824708	LMF2	24.14928244
DHRS7B	2.314528267	OSBPL3	24.3938369
IPO5	3.601853017	UQCRC2	24.54005743
SNRNP200	3.628937447	TALDO1	28.19632158
SND1	3.658741159	MED20	28.31508158
EIF3A	4.089054501	EZR	28.90554292
DDB1	4.206525736	YWHAB	31.17161516
HSPG2	4.306686412	CENPU	32.3507809
FGD6	4.458766508	SLC25A4	32.63073721
FLNB	4.69689811	CYB5R3	32.98043854
UTP20	4.77253981	PSMB5	34.80884536
VPS8	4.881711399	PEX11B	36.12220758
SRP54	5.017651843	CAPZA2	47.1375462
POLR1C	5.542300403	FTL	47.80156018
PHB	7.590819271	ARPC5	49.82976527
DBT	23.82994615		

### METTL3 interact with circCTTN through NOP2 and EIF3A

**Figure 4:**
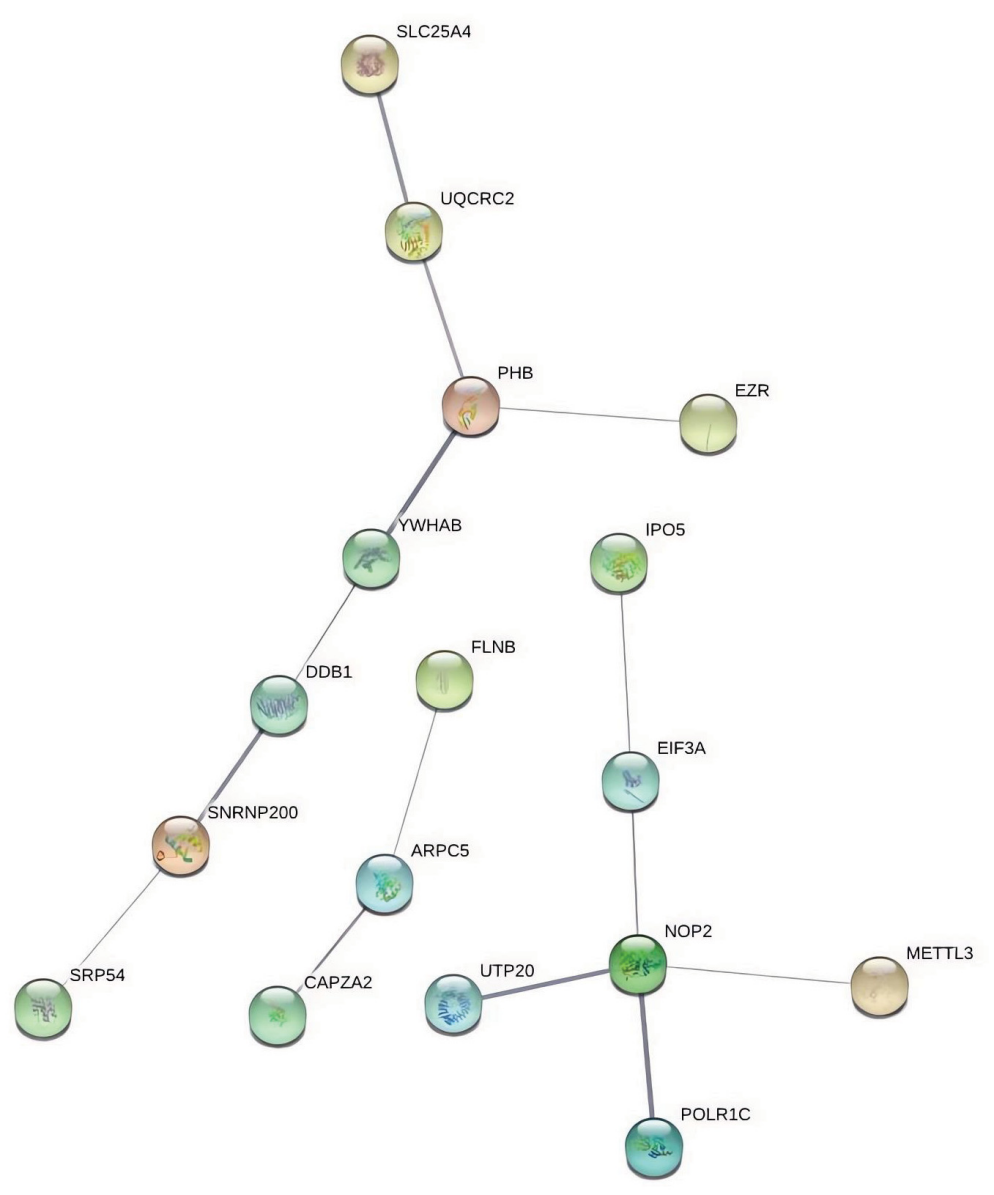
Identification of proteins potentially interacting with METTL3

**Figure 5:**
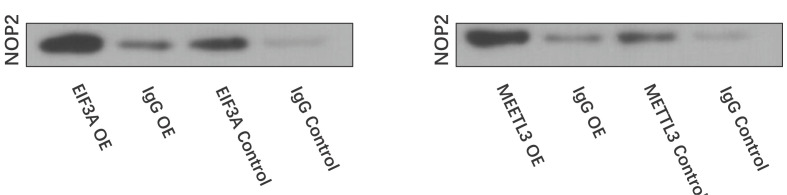
Co-IP and Western blotting of the interaction between NOP2 and EIF3A/METTL3

STRING database was used to analyze the interactions of the 31 pulled down proteins. Seventeen proteins interacted with each other (Figure 4). Co-IP showed that eukaryotic translation initiation factor 3 subunit A (EIF3A) and METTL3 both interacted with and nucleolar protein 2 (NOP2) directly (Figure 5), which indicated that METTL3 interacted with NOP2 and EIF3A directly, and METTL3 interacted with circCTTN through NOP2 and EIF3A. Staphylococcal nuclease domain-containing protein 1 (SND1) did not interact with other proteins, including EIF3A and METTL3.

## Discussion

Although hUCMSCs are ideal seed cells with multi-differentiation potential for bone tissue engineering, the exact regulatory mechanism of osteogenic differentiation of hUCMSCs remains elusive [38].

circRNA is a stable noncoding RNA with covalently closed loop structure [39], and plays an important role in various pathophysiological processes by regulating transcription, translation and coding proteins [14–17,40]. Many studies have reported that circRNAs act as miRNA sponges, by which they inhibit miRNA activity to regulate expression of miRNA target genes [41–44]. Our previous study determined the expression level of a critical noncoding RNA, circCTTN with a length of 258 bp derived from the chr11 CTTN (cortactin) gene exon 1-3, which was up-regulated in osteogenesis-induced hUCMSCs, and overexpression of circCTTN stimulated hUCMSC osteogenic differentiation [32]. This clearly demonstrated the close association between circCTTN and osteogenic differentiation of hUCMSCs. However, how circCTTN promoted osteogenic differentiation remains to be resolved.

As the most abundant internal modification of genes, m6A modification of circRNAs is been involved in various pathophysiological processes [21,45–47]. In the present study, we identified two potential m6A modification sites of circCTTN, which indicated that circCTTN could be methylated to regulate osteogenic differentiation of hUCMSCs.

METTL3, with a methyltransferase domain, can regulate mRNA translation, cell proliferation and apoptosis, cell migration and invasion, cell differentiation, and inflammatory response [48]. In the present study, we overexpressed METTL3 to explore its effect on m6A level of circCTTN and osteogenic differentiation of hUCMSCs. METTL3 overexpression promoted expression of osteogenesis-related genes and circCTTN, as well as the m6A levels of circCTTN at two predicted sites, circCTTN-54 and circCTTN-159. Previous studies have demonstrated that m6A modification of circRNA stabilizes circRNA to enhance the molecular sponge activity to target miRNAs [18,47,49]. Similarly, overexpression of METTL3 may increase the stability of circCTTN by up-regulating its m6A level, although this needs further investigation.

To study further the specific regulatory mechanism of METTL3 in m6A modification of circCTTN, RNA pulldown was performed using a biotin-labeled probe targeting circCTTN, followed by Western blotting and protein mass spectrometry to identify potential proteins involved in the m6A modification of circCTTN. Data from RNA pulldown assay indicated that circCTTN did not interact directly with METTL3, nor with the rest of the methyltransferase complex member proteins, such as METTL14, WTAP, and VIRMA. However, we identified two m6A readers (EIF3A and SND1) among the 31 proteins pulled-down by circCTTN-specific probe. m6A readers are proteins that recognize m6A sites, and they are able to bind to methylated RNA to confer downstream functions [24,25]. One of the m6A readers identified in the m6A modification in this study is SND1. Baquero Pérez et al. reported that the m6A modification of mRNA was critical for SND1 binding, which in turn increased the stability of the transcript [50]. SND1 is highly expressed in most cancer cells, and its expression level is associated with prognosis of patients [51]. Meng et al. demonstrated that expression of SND1 mRNA was markedly increased in colorectal tumor tissues, and SND1 altered m6A levels in colorectal cancer cell lines [52].

EIF3A, one of the core subunits of the translation initiation complex, is an m6A reader without YTH domain [53]. Meyer et al. reported that EIF3A can directly bind to mRNAs containing m6A in their 5′ UTR and initiate cap-independent translation for mRNAs [54]. Jin et al. indicated that YTHDF1 promoted YAP mRNA translation by interacting with EIF3A, and this activity was regulated by m6A modification [55]. However, YTH proteins recognize m6A through their specific YTH domain, while it remains unknown how EIF3A recognizes m6A and the protein domains necessary for the interaction [56]. Proliferation-associated NOP2 is a member of the NOP2/NSUN RNA-methyltransferase family, which can catalyze C5 methylation of RNA cytosine [57]. NOP2 is widely regarded as a marker for cancer aggressiveness [58]. Ma et al. reported that NOP2 promoted the growth of mouse fibroblasts and tumor formation [59]. In our study, we found that both EIF3A and NOP2 were pulled down together with circCTTN. Further bioinformatics analysis and Co-IP showed that NOP2 interacted with EIF3A and METTL3 directly, which indicated that EIF3A and NOP2 acted as a complex to mediate m6A modification of circCTTN by METTL3. Therefore, METTL3 up-regulates the m6A modification of circCTTN through interacting with EIF3A and NOP2 but not with SND1.

## Conclusions

This study presented a potential mechanism of m6A modification of a specific noncoding RNA (circCTTN) in hUCMSC osteogenic differentiation. Specifically, METTL3 promoted osteogenic differentiation of hUCMSCs by modifying the m6A sites and regulating the m6A level of circCTTN. Further analysis identified that EIF3A and NOP2 play a role in the modification process. METTL3 interacted with circCTTN through NOP2 and EIF3A. The findings of this study provide novel insights into the role of METTL3 and circCTTN in osteogenic differentiation of hUCMSCs.

## Supplementary Material

Supplementary Material S1-S5 and Figures

## Data Availability

The data that support the findings of this study are available from the corresponding author, upon reasonable request.

## Abbreviations

ALP: alkaline phosphatase
BMSC: bone marrow mesenchymal stem cell
Co-IP: co-immunoprecipitation
DEPC: diethyl pyrocarbonate
EIF3A: eukaryotic translation initiation factor 3 subunit A
hUCMSC: human umbilical cord mesenchymal stem cell
METTL3: methyltransferase-like 3
m6A: N6-methyladenosine
NOP2: nucleolar protein 2
qRT-PCR: quantitative real-time polymerase chain reaction
SND1: Staphylococcal nuclease domain-containing protein 1
WTAP: Wilms’ tumor 1-associated protein
