# Supplementary material for: METTL3 promotes osteogenic differentiation of human umbilical cord mesenchymal stem cells by up-regulating m6A modification of circCTTN
Source: Biosci Rep. 2024 Mar 12;44(3):BSR20231186. doi: 10.1042/BSR20231186 (PMC10932744; doi:10.1042/BSR20231186)
Supplement: Supplementary Material S1-S5 and Figures [file BSR-2023-1186_supp.pdf]

Supplementary Material 1

ALP staining

Figure 1 ALP staining of OE-METTL3 group

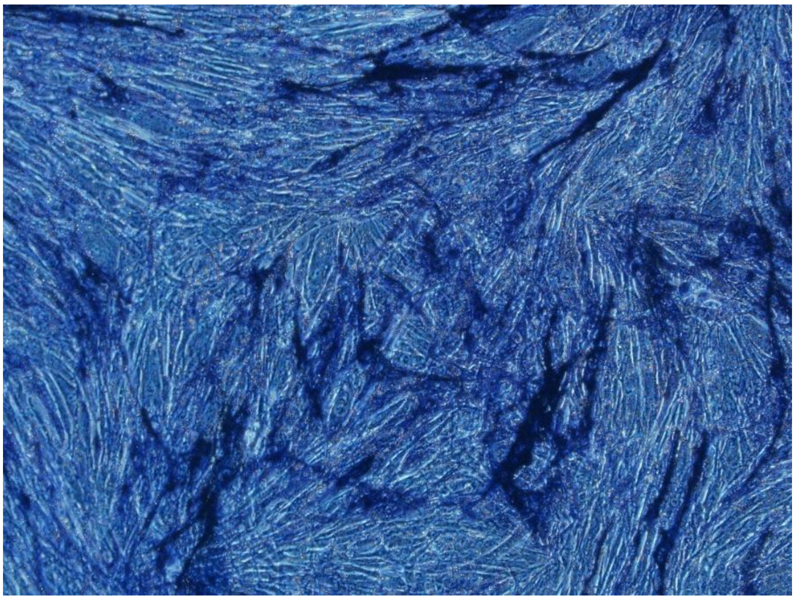

Figure 2 ALP staining of Control group

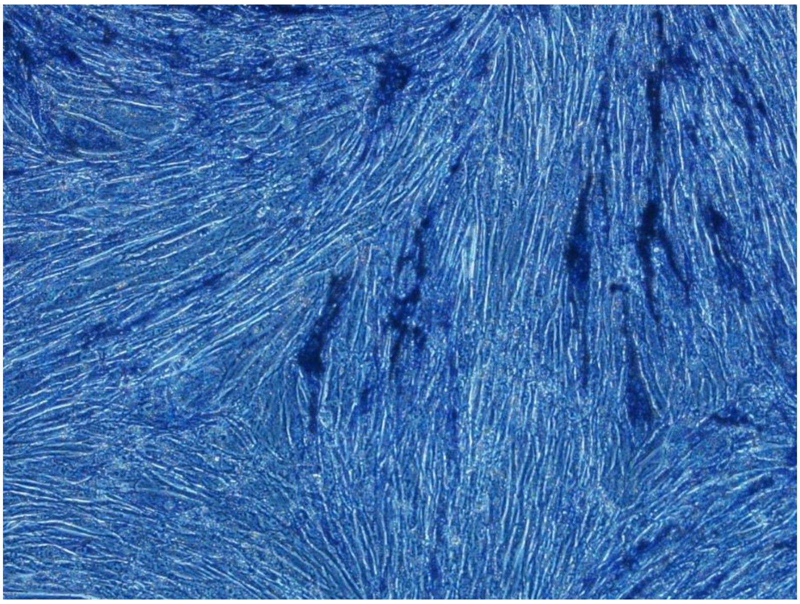

Supplementary Material 2

Cloud-Seq RNA QC Report

Total RNA QC Report

Table 1 RNA Quantification and Quality Assurance by NanoDrop ND-1000

Sample ID	Sample Name	OD260/280 Ratio	OD260/230 Ratio	Conc. (ng/μl)	Volume (μl)	Quanity (ug)	QC result
1	OE-METTL3 group (M group)	1.85	1.88	1184.13	157	185.91	**Pass**
2	Vector group (negative control group, NC group)	1.87	1.85	1151.41	157	180.77	**Pass**
3	Control group (C group)	1.84	1.86	1181.44	157	185.49	**Pass**

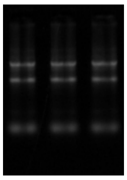

Figure 1 RNA Integrity and gDNA contamination test by Denaturing Agarose Gel Electrophoresis

*For spectrophotometer, the O.D. A260 /A280 ratio should be close to 2.0 for pure RNA (ratios between 1.8 and 2.1 are acceptable). The O.D. A260/A230 ratio should be more than 1.8.

Table 2 Primer Sequence

Gene	Gene Name	Primer	Sequence
Gene1	hsa_circ_0003376	1-Forward	GGAGACCGACCCTGATTTTG
		1-Reverse	CGCTTGGAGATAAAAGGCTGT
Reference	ACTB	11-Forward	GTGGATCAGCAAGCAGGAGT
		11-Reverse	AAAGCCATGCCAATCTCATC

Table 3 Result of ACTB

Name	Reporter	CT	Ct Mean	Ct SD
M	SYBR	18.599	18.567	0.029
	SYBR	18.542		
	SYBR	18.560		
NC	SYBR	18.460	18.452	0.016
	SYBR	18.433		
	SYBR	18.462		
C	SYBR	18.970	18.956	0.026
	SYBR	18.926		
	SYBR	18.973		

Table 4 Result of hsa_circ_0003376

Name	Reporter	CT	Ct Mean	Ct SD
M	SYBR	32.533	32.519	0.056
	SYBR	32.458		
	SYBR	32.568		
NC	SYBR	33.920	33.898	0.030
	SYBR	33.864		
	SYBR	33.909		
C	SYBR	34.609	34.609	0.017
	SYBR	34.591		
	SYBR	34.625		

Summary reports

Using ACTB as the reference and M group as the baseline，The results of are corrected as follows：

Table 5 The results of hsa_circ_0003376

Group	Sample Name	reference	hsa_circ_0003376	(hsa_circ_0003376) Ct- (ACTB) Ct	[(hsa_circ_0003376)Ct-(ACTB)Ct]NC or C-[(hsa_circ_0003376)Ct-(ACTB)Ct]M	$2^{-\Delta\Delta CT}$
M	M	18. 567	32. 519	13. 952	0. 000	1. 000
NC	NC	18. 452	33. 898	15. 446	1. 494	0. 355
C	C	18. 956	34. 609	15. 652	1. 700	0. 308
M/NC						2. 817
M/C						3. 250
NC/C						1. 154

Amplification curve

Figure 2 Amplification curve of ACTB

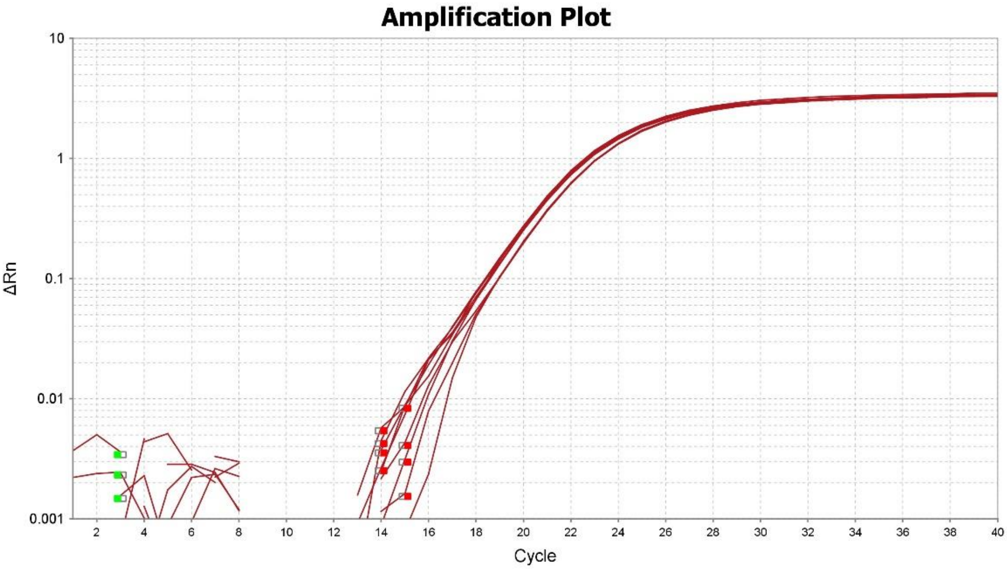

Figure 3 Amplification curve of hsa_circ_0003376

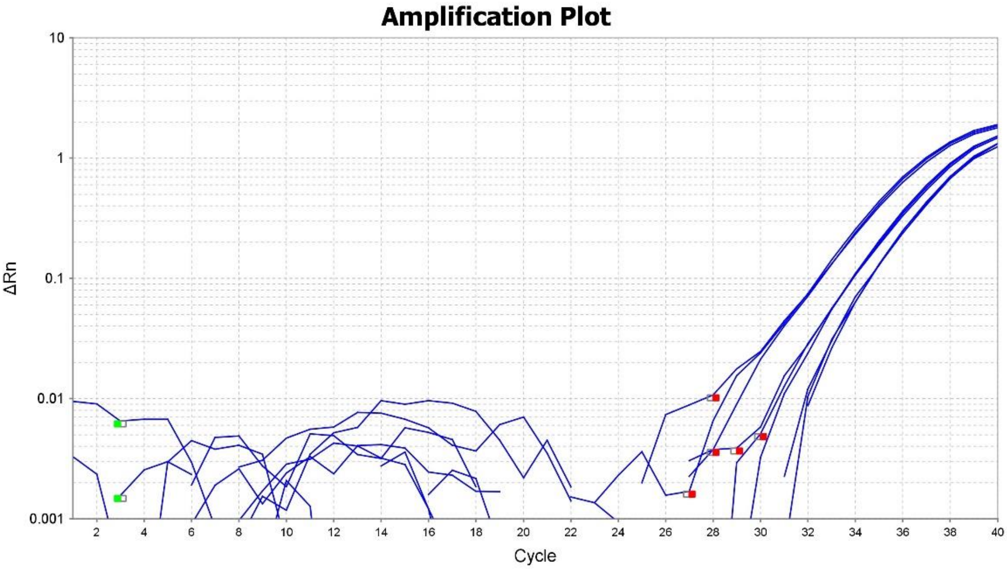

Melting curve

Figure 4 Melting curve of ACTB

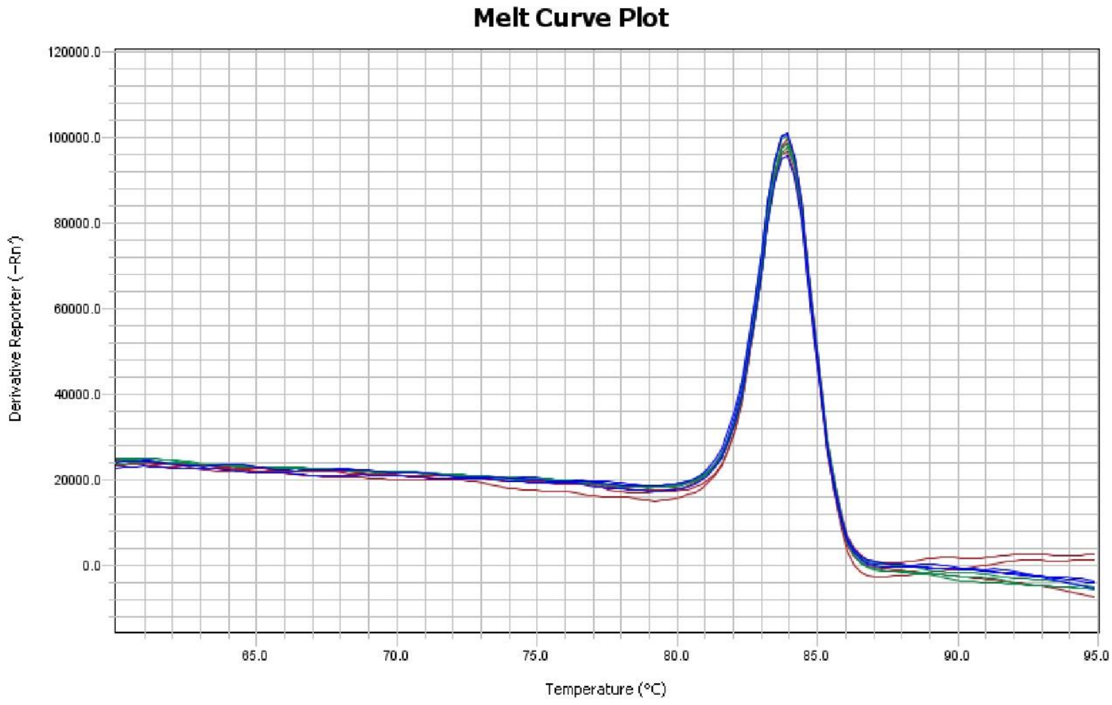

Figure 5 Melting curve of hsa_circ_0003376

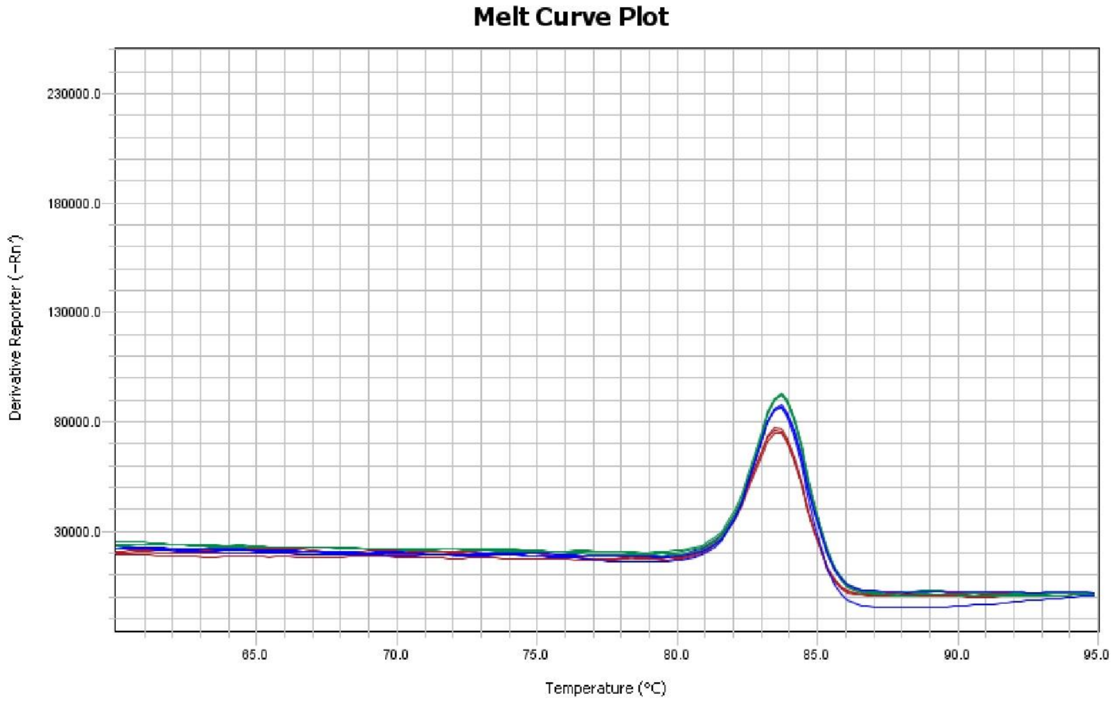

Supplementary Material 3

circCTTN sequence

ACGGAAUCAGUCCCCAAUGCCUGGAAAUUCCUCAUUGGAUUACUGUGUUUUAA**A**CAGAA
UUUCGUGAACAGCCUUUUAUCUCCAAGCGGAAAGAAAGAUGUGGAAAGCUUCAGCAGGC
CACGCUGUGUCCAUCGCCCAGGAUGACGCGGGGGCCGAUG**A**CUGGGAGACCGACCCUGAU
UUUGUGAAUGAUGUGAGUGAGAAGGAGCAAAGAUGGGGUGCCAAGACGGUGCAGGGCU
CCGGGCACCAGGAGCAUAUCAA

RMBase v.2.0

http://rna.sysu.edu.cn/rmbase/

Figure 1 RMBase v.2.0 prediction results

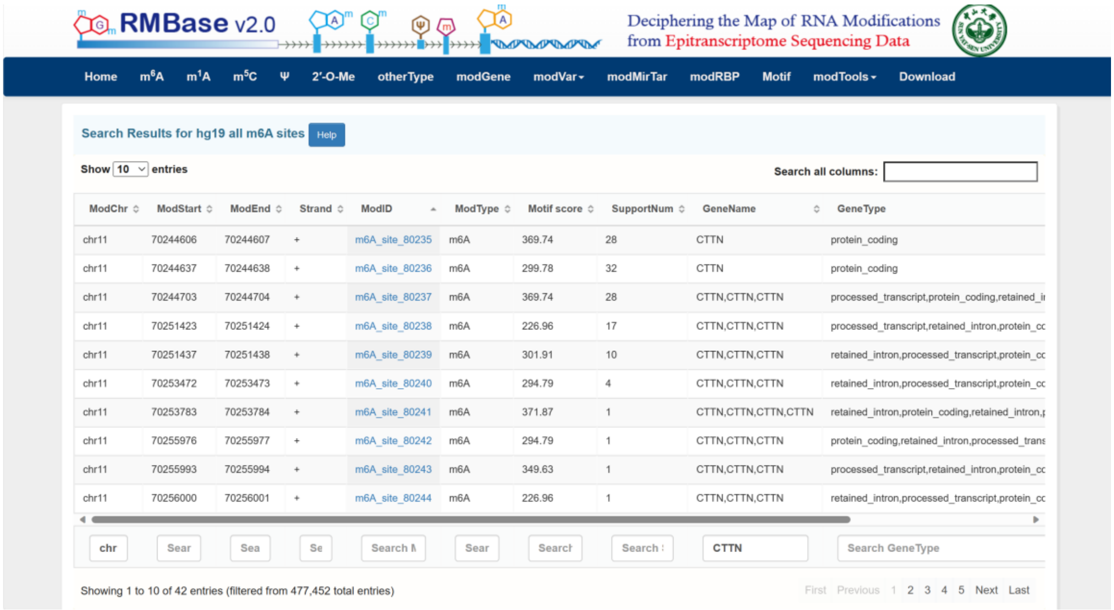

ModChr	ModStart	ModEnd	Strand	ModID	ModType	Motif score	SupportNum	GeneName	GeneType
chr11	70244606	70244607	+	m6A_site_80235	m6A	369.74	28	CTTN	protein_coding
chr11	70244637	70244638	+	m6A_site_80236	m6A	299.78	32	CTTN	protein_coding
chr11	70244703	70244704	+	m6A_site_80237	m6A	369.74	28	CTTN,CTTN,CTTN	processed_transcript,protein_coding,retained_i
chr11	70251423	70251424	+	m6A_site_80238	m6A	226.96	17	CTTN,CTTN,CTTN	processed_transcript,retained_intron,protein_cc
chr11	70251437	70251438	+	m6A_site_80239	m6A	301.91	10	CTTN,CTTN,CTTN	retained_intron,processed_transcript,protein_cc
chr11	70253472	70253473	+	m6A_site_80240	m6A	294.79	4	CTTN,CTTN,CTTN	retained_intron,processed_transcript,protein_cc
chr11	70253783	70253784	+	m6A_site_80241	m6A	371.87	1	CTTN,CTTN,CTTN,CTTN	retained_intron,protein_coding,retained_intron,p
chr11	70255976	70255977	+	m6A_site_80242	m6A	294.79	1	CTTN,CTTN,CTTN	protein_coding,retained_intron,processed_trans
chr11	70255993	70255994	+	m6A_site_80243	m6A	349.63	1	CTTN,CTTN,CTTN	processed_transcript,retained_intron,protein_cc
chr11	70256000	70256001	+	m6A_site_80244	m6A	226.96	1	CTTN,CTTN,CTTN	retained_intron,processed_transcript,protein_cc

RMBase v2.0 — Deciphering the Map of RNA Modifications from Epitranscriptome Sequencing Data

Home | m6A | m1A | m5C | Ψ | 2'-O-Me | otherType | modGene | modVar | modMirTar | modRBP | Motif | modTools | Download

Search Results for hg19 all m6A sites Help

Show 10 entries — Search all columns:

ModChr	ModStart	ModEnd	Strand	ModID	ModType	Motif score	SupportNum	GeneName	GeneType
chr11	70271488	70271489	+	m6A_site_80266	m6A	296.92	24	CTTN,CTTN,CTTN	processed_transcript,protein_coding,retained_i
chr11	70269045	70269046	+	m6A_site_80265	m6A	344.64	5	CTTN,CTTN,CTTN	processed_transcript,protein_coding,retained_i
chr11	70267749	70267750	+	m6A_site_80264	m6A	344.64	1	CTTN,CTTN,CTTN	retained_intron,protein_coding,retained_intron
chr11	70267626	70267627	+	m6A_site_80263	m6A	419.59	6	CTTN,CTTN,CTTN,CTTN	retained_intron,protein_coding,retained_intron,p
chr11	70267583	70267584	+	m6A_site_80262	m6A	344.64	7	CTTN,CTTN,CTTN,CTTN	protein_coding,protein_coding,retained_intron,r
chr11	70266556	70266557	+	m6A_site_80261	m6A	296.92	4	CTTN,CTTN	protein_coding,retained_intron
chr11	70266551	70266552	+	m6A_site_80260	m6A	296.92	4	CTTN,CTTN	protein_coding,retained_intron
chr11	70266543	70266544	+	m6A_site_80259	m6A	296.92	4	CTTN,CTTN	protein_coding,retained_intron
chr11	70266505	70266506	+	m6A_site_80258	m6A	344.64	4	CTTN,CTTN	protein_coding,retained_intron
chr11	70266437	70266438	+	m6A_site_80257	m6A	226.96	3	CTTN,CTTN	protein_coding,retained_intron
chr	Sear	Sea	Se	Search M	Sear	Search	Search :	CTTN	Search GeneType

Showing 11 to 20 of 42 entries (filtered from 477,452 total entries) — First Previous 1 2 3 4 5 Next Last

RMBase v2.0 — Deciphering the Map of RNA Modifications from Epitranscriptome Sequencing Data

Home | m6A | m1A | m5C | Ψ | 2'-O-Me | otherType | modGene | modVar | modMirTar | modRBP | Motif | modTools | Download

Search Results for hg19 all m6A sites Help

Show 10 entries — Search all columns:

ModChr	ModStart	ModEnd	Strand	ModID	ModType	Motif score	SupportNum	GeneName	GeneType
chr11	70266038	70266039	+	m6A_site_80256	m6A	226.96	6	CTTN,CTTN	protein_coding,retained_intron
chr11	70265893	70265894	+	m6A_site_80255	m6A	371.87	19	CTTN,CTTN	protein_coding,retained_intron
chr11	70263169	70263170	+	m6A_site_80254	m6A	296.92	11	CTTN,CTTN	protein_coding,retained_intron
chr11	70263118	70263119	+	m6A_site_80253	m6A	344.64	5	CTTN,CTTN	protein_coding,retained_intron
chr11	70263065	70263066	+	m6A_site_80252	m6A	301.91	2	CTTN,CTTN,CTTN	retained_intron,retained_intron,protein_coding
chr11	70261798	70261799	+	m6A_site_80251	m6A	344.64	1	CTTN,CTTN	protein_coding,retained_intron
chr11	70260744	70260745	+	m6A_site_80250	m6A	371.87	1	CTTN,CTTN	protein_coding,retained_intron
chr11	70260702	70260703	+	m6A_site_80249	m6A	419.59	1	CTTN,CTTN	retained_intron,protein_coding
chr11	70260676	70260677	+	m6A_site_80248	m6A	274.68	1	CTTN,CTTN	protein_coding,retained_intron
chr11	70256052	70256053	+	m6A_site_80247	m6A	294.79	1	CTTN,CTTN,CTTN	retained_intron,processed_transcript,protein_coding
chr	Sear	Sea	Se	Search M	Sear	Search	Search :	CTTN	Search GeneType

Showing 21 to 30 of 42 entries (filtered from 477,452 total entries) — First Previous 1 2 3 4 5 Next Last

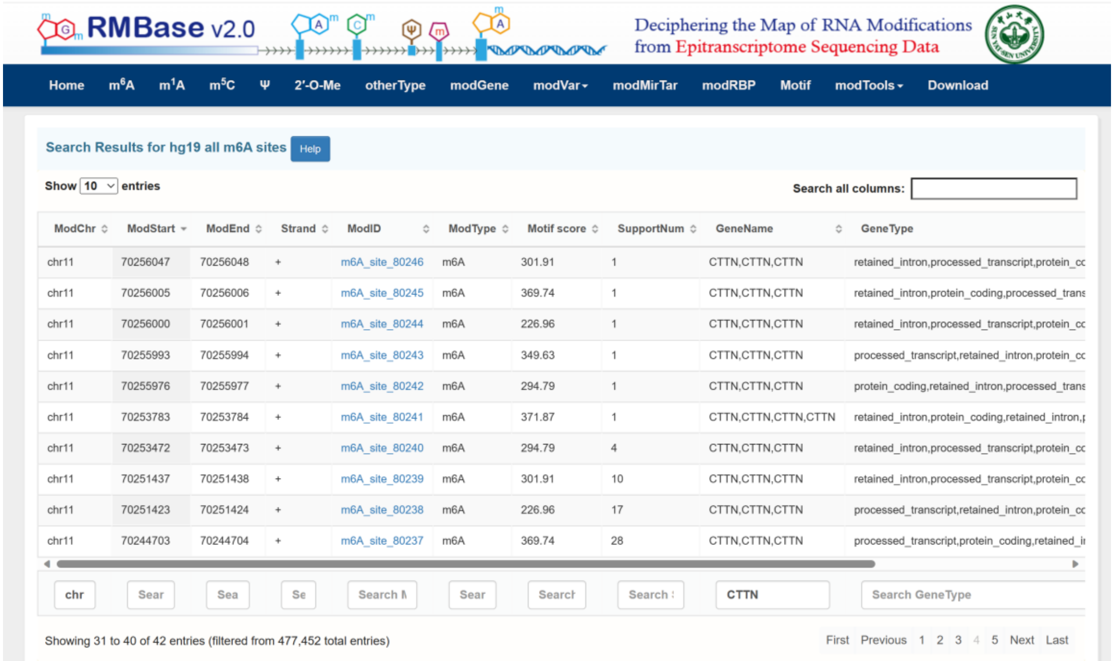

Search Results for hg19 all m6A sites Help

Show 10 entries Search all columns:

ModChr	ModStart	ModEnd	Strand	ModID	ModType	Motif score	SupportNum	GeneName	GeneType
chr11	70256047	70256048	+	m6A_site_80246	m6A	301.91	1	CTTN,CTTN,CTTN	retained_intron,processed_transcript,protein_cc
chr11	70256005	70256006	+	m6A_site_80245	m6A	369.74	1	CTTN,CTTN,CTTN	retained_intron,protein_coding,processed_trans
chr11	70256000	70256001	+	m6A_site_80244	m6A	226.96	1	CTTN,CTTN,CTTN	retained_intron,processed_transcript,protein_cc
chr11	70255993	70255994	+	m6A_site_80243	m6A	349.63	1	CTTN,CTTN,CTTN	processed_transcript,retained_intron,protein_cc
chr11	70255976	70255977	+	m6A_site_80242	m6A	294.79	1	CTTN,CTTN,CTTN	protein_coding,retained_intron,processed_trans
chr11	70253783	70253784	+	m6A_site_80241	m6A	371.87	1	CTTN,CTTN,CTTN,CTTN	retained_intron,protein_coding,retained_intron,p
chr11	70253472	70253473	+	m6A_site_80240	m6A	294.79	4	CTTN,CTTN,CTTN	retained_intron,processed_transcript,protein_cc
chr11	70251437	70251438	+	m6A_site_80239	m6A	301.91	10	CTTN,CTTN,CTTN	retained_intron,processed_transcript,protein_cc
chr11	70251423	70251424	+	m6A_site_80238	m6A	226.96	17	CTTN,CTTN,CTTN	processed_transcript,retained_intron,protein_cc
chr11	70244703	70244704	+	m6A_site_80237	m6A	369.74	28	CTTN,CTTN,CTTN	processed_transcript,protein_coding,retained_ir
chr	Sear	Sea	Se	Search M	Sear	Search	Search :	CTTN	Search GeneType

Showing 31 to 40 of 42 entries (filtered from 477,452 total entries) First Previous 1 2 3 4 5 Next Last

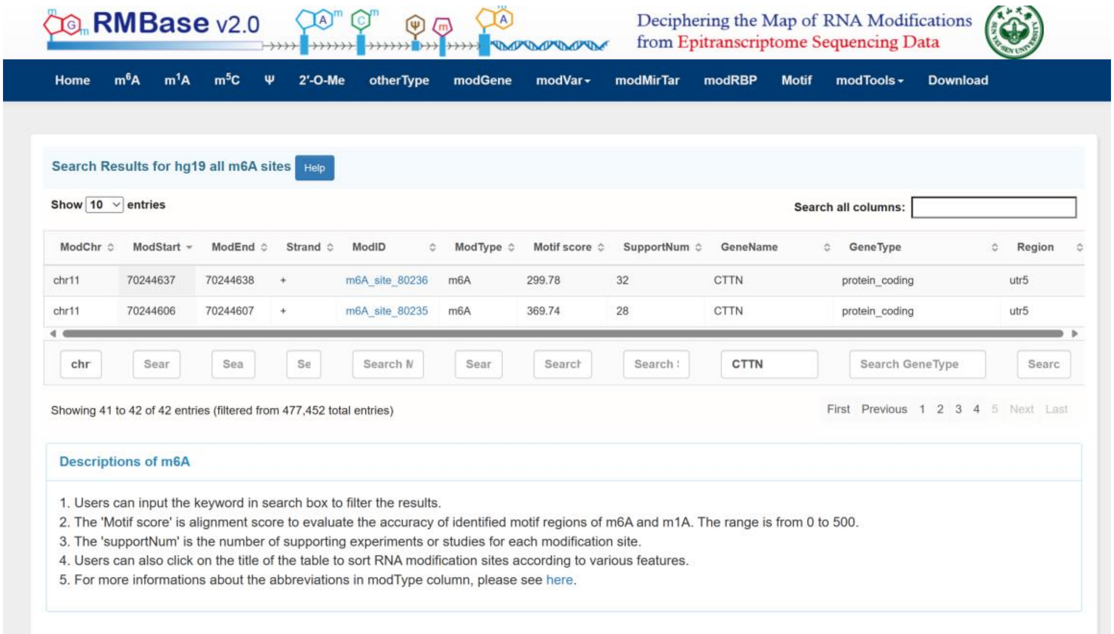

Search Results for hg19 all m6A sites Help

Show 10 entries Search all columns:

ModChr	ModStart	ModEnd	Strand	ModID	ModType	Motif score	SupportNum	GeneName	GeneType	Region
chr11	70244637	70244638	+	m6A_site_80236	m6A	299.78	32	CTTN	protein_coding	utr5
chr11	70244606	70244607	+	m6A_site_80235	m6A	369.74	28	CTTN	protein_coding	utr5
chr	Sear	Sea	Se	Search M	Sear	Search	Search :	CTTN	Search GeneType	Searc

Showing 41 to 42 of 42 entries (filtered from 477,452 total entries) First Previous 1 2 3 4 5 Next Last

Descriptions of m6A

1. Users can input the keyword in search box to filter the results.
2. The 'Motif score' is alignment score to evaluate the accuracy of identified motif regions of m6A and m1A. The range is from 0 to 500.
3. The 'supportNum' is the number of supporting experiments or studies for each modification site.
4. Users can also click on the title of the table to sort RNA modification sites according to various features.
5. For more informations about the abbreviations in modType column, please see here.

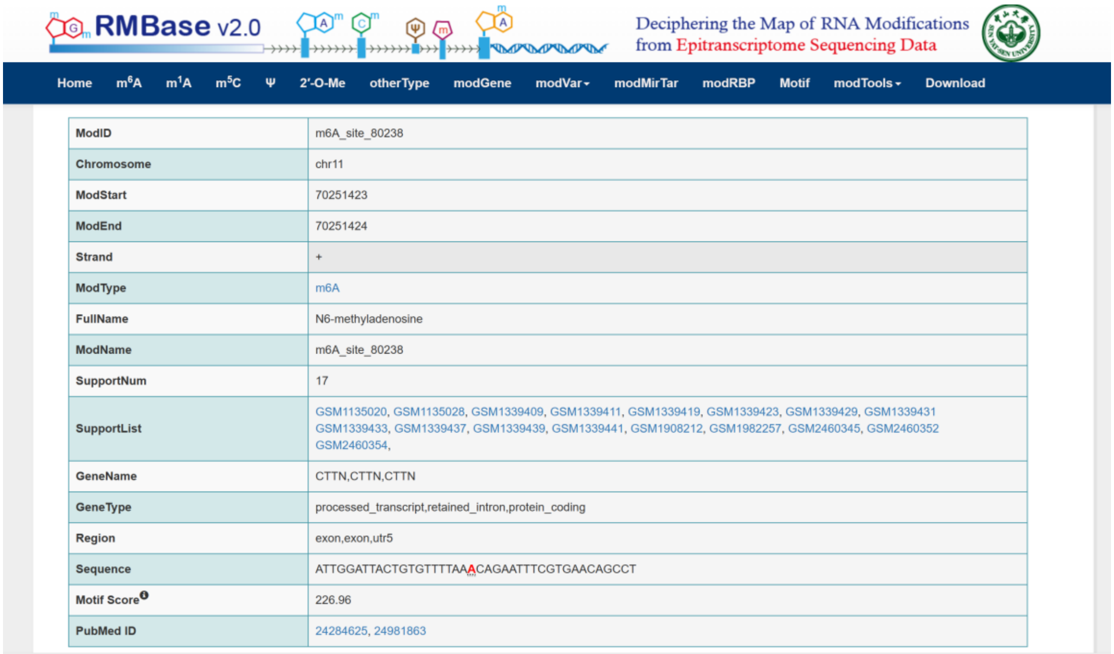

ModID	m6A_site_80238
Chromosome	chr11
ModStart	70251423
ModEnd	70251424
Strand	+
ModType	m6A
FullName	N6-methyladenosine
ModName	m6A_site_80238
SupportNum	17
SupportList	GSM1135020, GSM1135028, GSM1339409, GSM1339411, GSM1339419, GSM1339423, GSM1339429, GSM1339431 GSM1339433, GSM1339437, GSM1339439, GSM1339441, GSM1908212, GSM1982257, GSM2460345, GSM2460352 GSM2460354,
GeneName	CTTN,CTTN,CTTN
GeneType	processed_transcript,retained_intron,protein_coding
Region	exon,exon,utr5
Sequence	ATTGGATTACTGTGTTTTAAACAGAATTTCGTGAACAGCCT
Motif Score	226.96
PubMed ID	24284625, 24981863

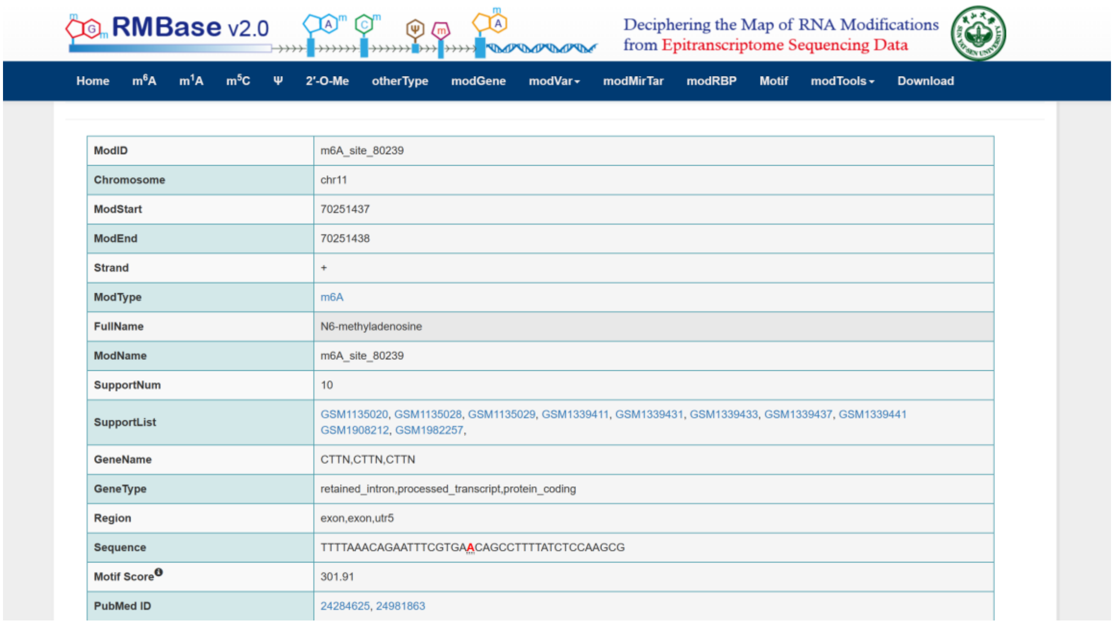

ModID	m6A_site_80239
Chromosome	chr11
ModStart	70251437
ModEnd	70251438
Strand	+
ModType	m6A
FullName	N6-methyladenosine
ModName	m6A_site_80239
SupportNum	10
SupportList	GSM1135020, GSM1135028, GSM1135029, GSM1339411, GSM1339431, GSM1339433, GSM1339437, GSM1339441 GSM1908212, GSM1982257,
GeneName	CTTN,CTTN,CTTN
GeneType	retained_intron,processed_transcript,protein_coding
Region	exon,exon,utr5
Sequence	TTTTAAACAGAATTTCGTGAACAGCCTTTTATCTCCAAGCG
Motif Score	301.91
PubMed ID	24284625, 24981863

BERMP

http://www.bioinfogo.org/bermp.

Figure 2 BERMP prediction results

BERMP prediction result

The text format:

==

Gene	Position	Sequence context	Score	Prediction
	54	AUUGGAUUACUGUGUUUUAAACAGAAUUUCGUGAACAGCCU	0.346479	High confidence
	68	UUUUAAACAGAAUUUCGUGAACAGCCUUUUAUCUCCAAGCG	0.180180	Moderate confidence
	159	GGAUGACGCGGGGGCCGAUGACUGGGAGACCGACCCUGAUU	0.086928	Low confidence
	167	CGGGGGCCGAUGACUGGGAGACCGACCCUGAUUUUGUGAAU	0.070198	No

SRAMP

http://www.cuilab.cn/sramp/.

Figure 3 SRAMP prediction results

Position

#	Position	Sequence context	Structural context	Local structure visualization	Score(binary)	Score(knn)	Score(spectrum)	Score(combined)	Decision
1	159	CAGGA UGACG CGGGG<br>GCCGA UGACU GGGAG<br>ACCGA CCCUG AUUUU	N/A	N/A	0.570	0.722	0.685	0.623	m^6A site (**High** confidence)

Supplementary Material 4

Cloud-Seq RNA QC Report

1. Total RNA QC Report

Table 1 RNA Quantification and Quality Assurance by NanoDrop ND-1000

Sample ID	Sample Name	OD260/280 Ratio	OD260/230 Ratio	Conc. (ng/μl)	Volume (μl)	Quanity (ug)	QC result
1	OE-METTL3 group (M group)	1.85	1.88	1184.13	157	185.91	**Pass**
2	Vector group (negative control group, NC group)	1.87	1.85	1151.41	157	180.77	**Pass**
3	Control group (C group)	1.84	1.86	1181.44	157	185.49	**Pass**

Figure 1 RNA Integrity and gDNA contamination test by Denaturing Agarose Gel Electrophoresis

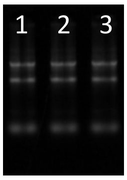

*For spectrophotometer, the O.D. A260 /A280 ratio should be close to 2.0 for pure RNA (ratios between 1.8 and 2.1 are acceptable). The O.D. A260/A230 ratio should be more than 1.8.

Table 2 Primer Sequence

Gene	Gene name	Primer	Sequence
Gene1	hsa_circ_0003376-1424	1-Forward	AATCAGTCCCCAATGCCTGG
		1-Reverse	GTCATCCTGGGCGATGGAC
Gene2	hsa_circ_0003376-159	2-Forward	AGCGGAAAGAAAGATGTGGA
		2-Reverse	CAAAATCAGGGTCGGTCTCC

Table 3 Me-RIP result of hsa_circ_0003376-54

Sample Name	Reporter		Ct	Ct Mean	Ct SD		Ct	Ct Mean	Ct SD
M	SYBR	IP	29.517	29.476	0.087	Input	31.100	31.136	0.055
	SYBR		29.376				31.200		
	SYBR		29.535				31.109		
NC	SYBR	IP	30.709	30.715	0.005	Input	30.587	30.585	0.017
	SYBR		30.719				30.567		
	SYBR		30.717				30.602		

**C**	SYBR	IP	30.647	30.660	0.015	Input	30.208	30.193	0.021
	SYBR		30.677				30.169		
	SYBR		30.655				30.200		

Table 4 Me-RIP result of hsa_circ_0003376-159

**Sample Name**	**Reporter**		**Ct**	**Ct Mean**	**Ct SD**		**Ct**	**Ct Mean**	**Ct SD**
**M**	SYBR	IP	28.640	28.650	0.027	Input	29.729	29.737	0.043
	SYBR		28.630				29.698		
	SYBR		28.680				29.782		
**NC**	SYBR	IP	30.104	30.158	0.060	Input	29.791	29.849	0.056
	SYBR		30.223				29.902		
	SYBR		30.147				29.854		
**C**	SYBR	IP	29.926	29.974	0.059	Input	29.692	29.603	0.079
	SYBR		30.040				29.540		
	SYBR		29.956				29.577		

Summary reports

Table 5 hsa_circ_0003376-54 result

**Group**	**Sample Name**	**Ct**		**%(IP/Input)**
		IP	Input	
M	M	29.476	31.136	17.56

NC	NC	30.715	30.585	5.08
C	C	30.660	30.193	4.02
M/NC				3.46
M/C				4.37
NC/C				1.26

Table 6 hsa_circ_0003376-159 results

Group	Sample Name	Ct		%(IP/Input)
		IP	Input	
M	M	28.650	29.737	11.80
NC	NC	30.158	29.849	4.48
C	C	29.974	29.603	4.30
M/NC				2.63
M/C				2.75
NC/C				1.04

Amplification curve

Figure 2 Amplification curve of hsa_circ_0003376-54

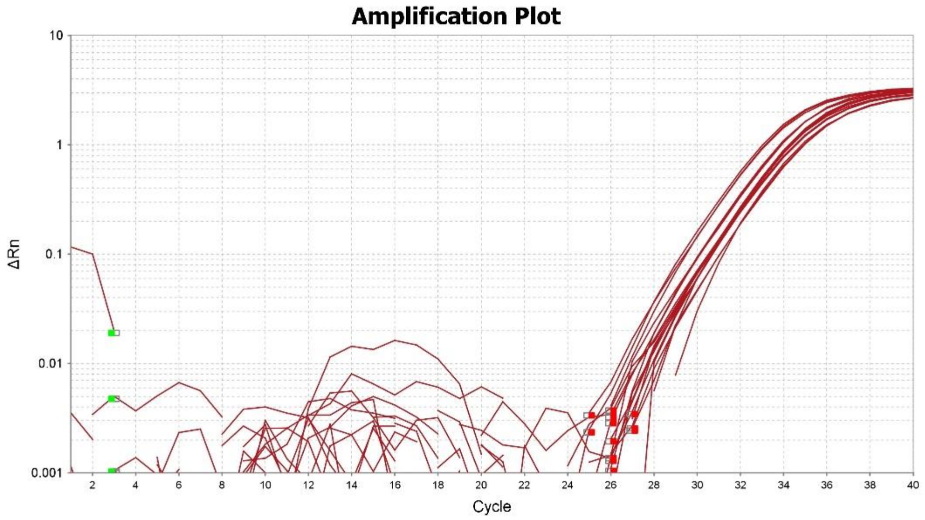

Figure 3 Amplification curve of hsa_circ_0003376-159

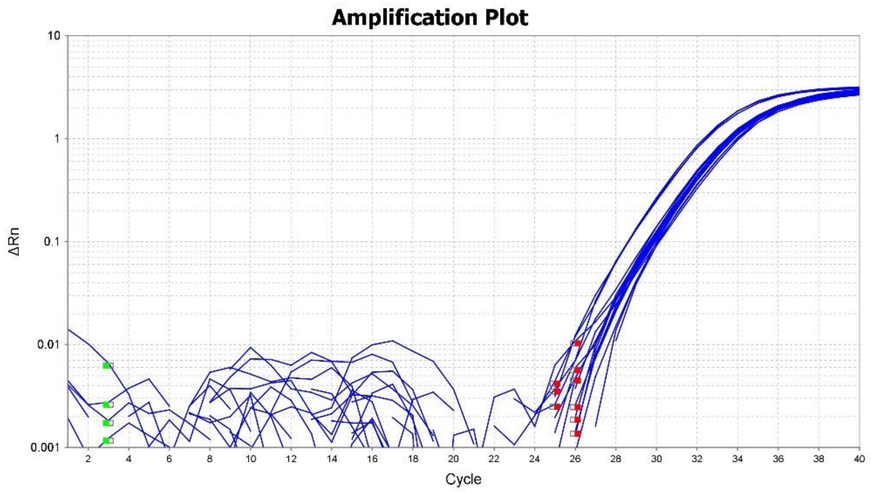

Melting curve

Figure 4 Melting curve of hsa_circ_0003376-54

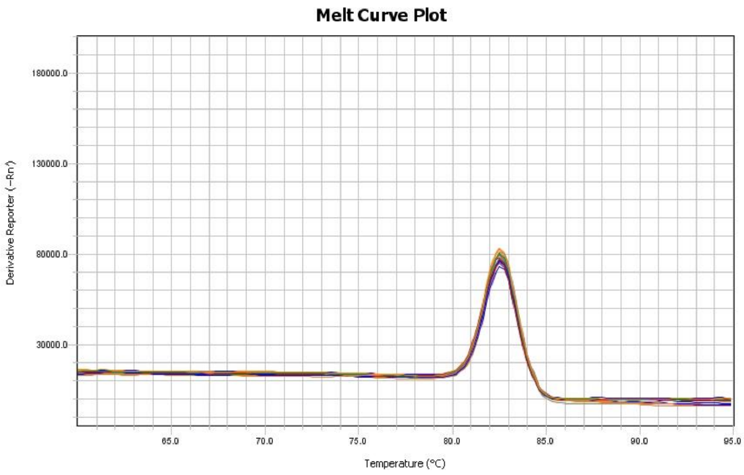

Figure 5 Melting curve of hsa_circ_0003376-159

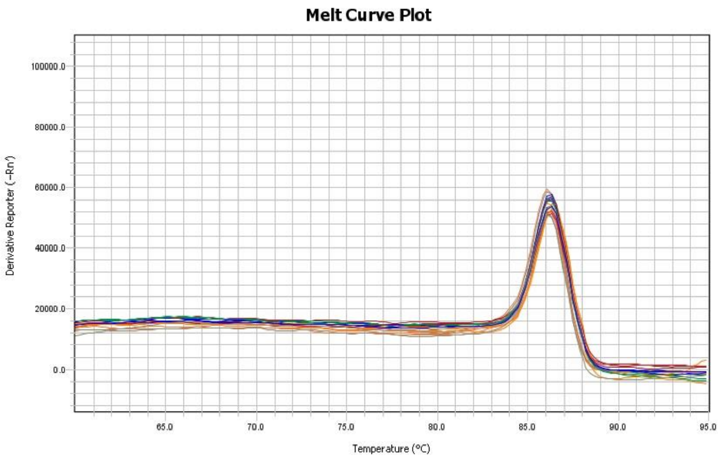

Supplementary Material 5

Table 1. Mass spectrometry of potential proteins pulled down by probes specific for circCTTN, which were upregulated significantly compared to the negative probe group (P<0.05).

Gene names	Fold Change	Regulation	hUCMSCs+	hUCMSCs-	hUCMSCs+	hUCMSCs-
IPO5	3.601853	up	25700	77587	4.7936338	2.9448945
POLR1C	5.5423004	up	238150	95739	5.4610294	2.9905444
ARPC5	49.829765	up	4036500	0	6.6389359	1
FLNB	4.6968981	up	129820	105350	5.2430256	3.0113173
SNRNP200	3.6289374	up	41916	133340	4.9220274	3.0624802

UTP20	4.7725398	up	143740	115910	5.2868177	3.0320605
PEX11B	36.122208	up	1430000	0	6.1748142	1
CYB5R3	32.980439	up	1020900	0	6.0435387	1
FTL	47.80156	up	3520800	0	6.5789858	1
DBT	23.829946	up	329960	0	5.5747038	1
SLC25A4	32.630737	up	981350	0	6.0281597	1
EZR	28.905543	up	672280	0	5.8532743	1
UQCRC2	24.540057	up	365780	0	5.6170667	1
PSMB5	34.808845	up	1267500	0	6.1213821	1
YWHAB	31.171615	up	861800	0	5.962161	1
PHB	7.5908193	up	844080	112780	5.9503716	3.026116
TALDO1	28.196322	up	601920	0	5.8174351	1
NOP2	2.1808247	up	3576300	350920	6.5859032	5.4610294
CAPZA2	47.137546	up	3336100	0	6.5588048	1
SRP54	5.0176518	up	192690	136550	5.3946582	3.0676459
HSPG2	4.3066864	up	37532	36959	4.8904384	2.7838601
EIF3A	4.0890545	up	41185	58995	4.9171749	2.8854076
DDB1	4.2065257	up	74713	100530	5.073777	3.0011478
DHRS7B	2.3145283	up	1303200	166600	6.1327456	4.9220274
FGD6	4.4587665	up	85606	81176	5.1113585	2.9547138
CENPU	32.350781	up	972960	0	6.0157286	1
SND1	3.6587412	up	36690	105380	4.8827265	3.0113791
VPS8	4.8817114	up	103460	61039	5.1801907	2.8928037
LMF2	24.149282	up	342030	0	5.5939084	1
OSBPL3	24.393837	up	355010	0	5.6084448	1
MED20	28.315082	up	610330	0	5.8234988	1
